# A hyperspectral imaging and machine learning approach for rapid and non-invasive diagnosis of cassava bacterial blight

**DOI:** 10.3389/fpls.2025.1707646

**Published:** 2026-01-26

**Authors:** Ian Carlos Bispo Carvalho, Luciellen da Costa Ferreira, Ana Régia de Mendonça Neves, Alice Maria Silva Carvalho, Henrique Póvoa Rodrigues Lima, Maurício Rossato

**Affiliations:** 1 Universidade de Brasília, Brasília, Distrito Federal, Brazil; 2 Instituto Federal de Brasília (IFB), Eixo de Informação e Comunicação, Distrito Federal, Brasília, Brazil

**Keywords:** bacterial blight, HSI, plant disease, plant physiology, Xanthomonas

## Abstract

This study explores the use of hyperspectral imaging (HSI) combined with machine learning to detect physiological alterations in cassava leaves caused by *Xanthomonas phaseoli* pv. *manihotis* (Xpm), a bacterial plant disease that causes significant yield losses worldwide. Therefore, the use of hyperspectral images associated with machine learning can provide information rapidly and accurately, aiming to support decision-making. HSI captures spectral data that reflects biochemical changes in infected plant tissues. An image set of cassava healthy and symptomatic leaves (402 and 450, respectively) were imaged using a hyperspectral camera across wavelengths from 400 to 1000 nm, with image calibration and spectral normalization to improve data quality. Spectral parameters, such as mean reflectance and spectral differences (healthy vs. infected), were analyzed. Six machine learning models were tested for classification: Decision Tree (DT), Random Forest (RF), Support Vector Machine (SVM), K-Nearest Neighbors (KNN), Extreme Gradient Boosting (XGBoost), and Multi-Layer Perceptron (MLP). SVM performed best, achieving the highest accuracy (91.41%), followed by MLP (87.89%), XGBoost (79.69%), and RF (77.34%). DT and KNN had the lowest accuracy (71.88% and 70.31%, respectively). The results suggest that HSI, particularly when combined with SVM, offers a rapid and accurate method for diagnosing cassava bacterial blight, with potential for large-scale field applications.

## 1 Introduction

Cassava bacterial blight, caused by *Xanthomonas phaseoli* pv. *manihotis* (Xpm), is a major phytosanitary challenge that threatens cassava production globally. Occurring in nearly 50 countries (Taylor et al., 2017; Zárate-Chaves et al., 2021), this disease causes yield losses ranging from 30% to 90%. Without proper management, complete crop failure can occur within two to three growing cycles (Mansfield et al., 2012; Zárate-Chaves et al., 2021). Symptoms include translucent, water-soaked leaf spots that progress to necrosis and may cause wilting in cases of systemic infection.

Successful disease management depends on the grower’s ability to detect outbreaks in the field, thereby anticipating and mitigating the deleterious effects of the phytopathogenic agents. In recent decades, considerable progress has been made in developing non-invasive techniques for plant disease diagnosis, such as fluorescence spectroscopy, VNIR spectroscopy, fluorescence imaging, and hyperspectral imaging (Sankaran et al., 2010; Golhani et al., 2018). Among these, hyperspectral sensors have gained prominence for their efficiency in extracting diverse types of information from plant tissues (Ortenberg, 2018). These techniques have found widespread application in agriculture, including seed quality analysis (Feng et al., 2019; Ferreira et al., 2024) and soil assessment (Demattê et al., 2010).

Despite the promise of hyperspectral imaging, challenges remain in selecting optimal wavelengths and scaling this technology for practical use in large agricultural settings. Addressing these challenges often requires the application of machine learning algorithms to extract meaningful insights from the spectral data and develop effective disease management strategies (Feng et al., 2021; Zhang et al., 2020).

Recent studies have highlighted the potential of spectral data and machine learning to understand the behavior of plants under pathogen infection across different pathosystems, such as tomato bacterial blight (Abdulridha et al., 2020), rice bacterial blight (Zhang et al., 2025), bacterial blight disease in red kidney beans (Qiao et al., 2025) and cassava brown streak disease (Peng et al., 2022). Machine learning, a key area within artificial intelligence, employs computational algorithms that learn from input data to perform various tasks, such as classification or clustering. This approach is particularly well-suited for identifying parameters and trends in hyperspectral data (Dhakal et al., 2023).

Machine learning techniques can be broadly categorized into supervised and unsupervised learning. In supervised learning, predictive models are trained using labeled data, where each data point is associated with a known “ground truth”, either assigned by experts or verified experimentally. In contrast, unsupervised learning identifies patterns in unlabeled data, without predefined labels (Greener et al., 2022; Asnicar et al., 2024). Additionally, semi-supervised learning, which combines both labeled and unlabeled data, can optimize the process, especially when data labeling is costly (Greener et al., 2022). Various machine learning algorithms, such as Support Vector Machines (SVM), Random Forest (RF), have been widely used to build plant disease prediction models, each offering different performances depending on the dataset and the application (Ahmad et al., 2023; Omaye et al., 2024).

Despite advances in this field, a gap remains in the application of these techniques for the detection of Xpm in cassava plants. Therefore, this study aims to employ an innovative approach that combines hyperspectral imaging with machine learning to identify cassava plants infected with *Xpm*, thereby improving the diagnosis and management of cassava bacterial blight.

## 2 Materials and methods

### 2.1 *Xpm* inoculation on cassava plants

The bacterial inoculum (UnB 17 isolate) was prepared by streaking the bacteria into 523 culture medium (Kado and Heskett, 1970) and incubated at 28°C for 48 hours in a growth chamber. Typical colonies were transferred to new plates for another incubation period. A bacterial suspension was subsequently prepared in distilled water, and its concentration was measured and adjusted using a Shimadzu UV-1203 spectrophotometer (wavelength of 550 nm and absorbance of 0.350) to obtain a bacterial concentration of 10^8^ CFU/mL.

Variety BGMC 962 of cassava, commonly used in Brazil and considered susceptible to Xpm, was chosen for the experiment and propagated by cuttings. Eight-week-old cassava plants were inoculated by spraying the aerial parts with the bacterial suspension using a handheld sprayer until runoff. The inoculated plants were maintained in a moist chamber for three days and then transferred to a greenhouse with controlled temperature (26°C), where they were monitored for disease development over the study period. Mock inoculated plants were kept with the same conditions. Twenty days after inoculation, both healthy and symptomatic leaves from cassava plants, exhibiting varying degrees of disease severity, were harvested for hyperspectral imaging. The leaf harvesting started 20 days after inoculation and extended over a 30-day period. By the end of this period, a total of 852 leaves were collected, comprising 402 healthy leaves and 450 symptomatic leaves.

### 2.2 Image capture

**Figure 1:**
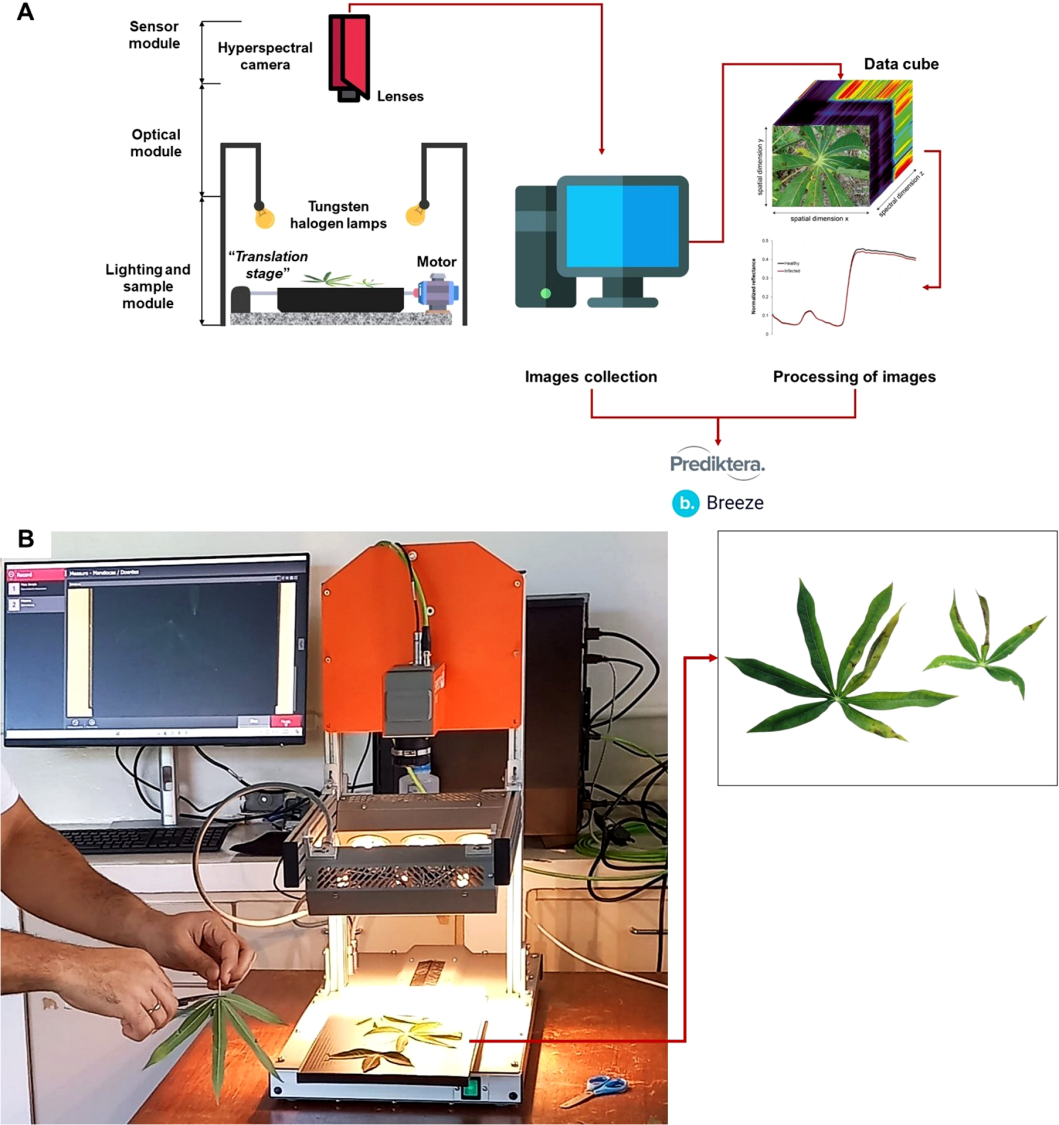
Schematic representation and setup of a benchtop hyperspectral imaging system for cassava leaf analysis. **(A)** Diagram of the hyperspectral imaging system components, including the FX10e camera, halogen lamps for illumination, and a motorized translation stage for sample movement. **(B)** Practical setup capturing cassava leaf samples under halogen lamp illumination.

The collection of hyperspectral imaging of cassava leaves was performed using an FX10e camera (Specim, Finland), capable of measuring reflectance within the 400–1000 nm spectral range, attached to a LabScanner (Specim, Finland) with six halogen lamps (**Figure 1**). Images were captured with Software Breeze v. 2024.1 (Prediktera, Sweden) also used for storage and initial processing of images. Each group of symptomatic leaves was distributed along the LabScanner tray for sample movement and proper hyperspectral image capture. An RGB image was also captured to serve as a reference for the normal appearance of the samples.

Before beginning the image capture, reference images for white and black were obtained with the camera shutter closed. The imaging was performed across all spectral bands within the capabilities of the equipment used for this project. To extract the true spectral response of each sample, the influence of the black and white reference images was removed, resulting in a calibrated image (IR) (Kim et al., 2001).

### 2.3 Hyperspectral image processing

Image segmentation and region of interest (ROI) selection were conducted using Breeze software. This process was designed to eliminate the background, enabling a clear view of the pixels representing cassava leaves (Baek et al., 2019). Following this, the spectral data was normalized using the Standard Normal Variate (SNV) method, which adjusted for spectral variations in the numerical data.

### 2.4 Spectral parameters

Three spectral parameters were used to identify wavelengths with significant differences between healthy and diseased leaves: (i) the mean reflectance values of cassava leaves infected with Xpm compared to healthy leaves; (ii) the spectral difference, calculated by subtracting the mean reflectance of healthy leaves from that of infected leaves at each wavelength; and (iii) sensitivity, determined by the ratio of the mean reflectance of diseased leaves to that of healthy leaves at each analyzed wavelength (Abdulridha et al., 2020). These parameters provided additional information to support the analysis, complementing the interpretation of the spectral data, and were not directly used in the modeling process.

### 2.5 Data analysis

**Figure 2:**
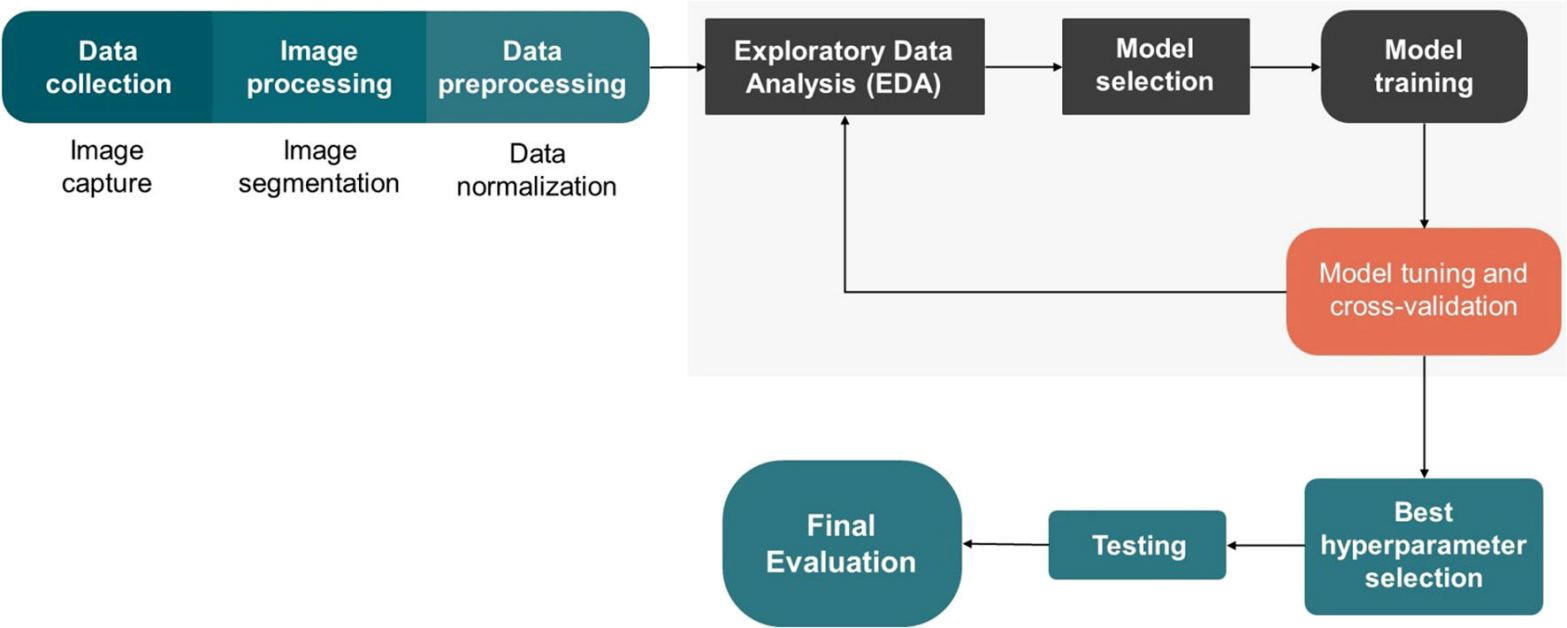
Workflow of the image classification process, including data collection, processing, hyperparameter optimization, training, testing, and performance evaluation.

The methodology for acquiring and analyzing hyperspectral data followed a structured approach, encompassing data collection, image processing, model selection, data preprocessing, model optimization, training, testing, and performance evaluation. The workflow of the process is illustrated in **Figure 2**. The analyses were conducted using Python, utilizing the following libraries: Optuna (Akiba et al., 2019), Scikit-learn (Pedregosa et al., 2011), XGBoost (Chen and Guestrin, 2016), Seaborn (Waskom et al., 2017), and Matplotlib (Hunter, 2007).

### 2.6 Classification methods and hyperparameter optimization

Six supervised learning methods were evaluated for the classification task: Decision Tree (DT), Random Forest (RF), Support Vector Machine (SVM), K-Nearest Neighbors (KNN), Extreme Gradient Boosting (XGBoost), and Multi-Layer Perceptron (MLP).

The processed dataset was partitioned into training (70%) and testing (30%) subsets using a stratified sampling approach in order to maintain the original class distribution in each subset. For each machine learning method, hyperparameter optimization was conducted using the Optuna library, with 5-fold stratified cross-validation used to evaluate each hyperparameter combination during training. The mean cross-validation score for each trial was computed to guide the optimization and ensure model robustness, helping to prevent overfitting. After optimization, the final pipeline for each method was trained on the training dataset using the best-found hyperparameters.

### 2.7 Model validation

The models’ performance was evaluated on the test set using the confusion matrix and the following metrics: accuracy, precision, recall, and F1 Score. These metrics are based on True Positive (TP), False Positive (FP), False Negative (FN), and True Negative (TN) values (Sujatha et al., 2021; Cunha et al., 2023), and are mathematically represented by (Equations 1, 2, 3 and 4):

(1) $$Accuracy=\;\frac{TP+TN}{TP+TN+FP+FN}$$

(2) $$Precision=\frac{TP}{TP+FP}$$

(3) $$Recall=\frac{TP}{TP+FN}$$

(4) $$F1\;Score=2\frac{Precision\;\times Recall}{Precision+Recall}$$

Additionally, the ROC-AUC (Receiver Operating Characteristic - Area Under the Curve) metric was calculated to evaluate the models’ ability to distinguish between classes (Gonçalves et al., 2014; Li, 2024).

## 3 Results

Healthy and infected cassava plants were evaluated, with symptomatic leaves exhibiting evolving symptoms from water-soaked spots to necrotic lesions. A total of 852 leaves, comprising 402 healthy and 450 symptomatic samples across various disease stages, were analyzed and divided into training and testing sets. For each leaf, 448 bands in the visible and near-infrared spectrum (400–1000 nm) were captured using a hyperspectral camera.

**Figure 3:**
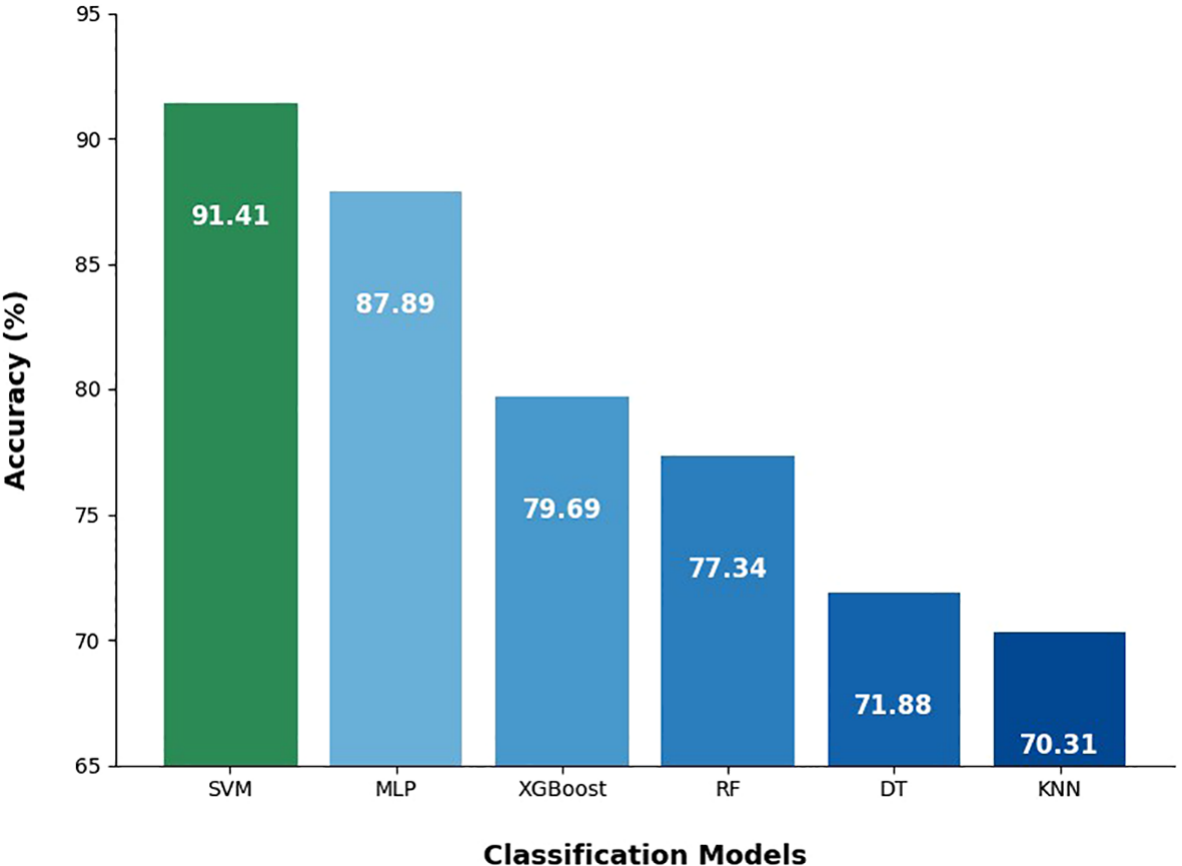
Accuracy results of classification methods differentiating healthy cassava leaves from those infected with Xpm. K-Nearest Neighbors (KNN); Decision Tree (DT); Random Forest (RF); Extreme Gradient Boosting (XGBoost); Multi-Layer Perceptron (MLP); Support Vector Machine (SVM).

The accuracy of the machine learning models applied to the spectral data of healthy cassava leaves and those infected by Xpm is presented in **Figure 3**. Among the evaluated models, SVM achieved the highest accuracy (91.41%), effectively distinguishing between healthy and infected leaf samples. The MLP model also yielded strong results (87.89%), indicating the neural network’s ability to detect subtle variations in the spectral data.

XGBoost and RF, both decision tree-based algorithms, attained accuracies of 79.69% and 77.34%, respectively, showing intermediate performance compared to SVM and MLP. DT achieved an accuracy of 71.88%, reflecting the model’s lower discriminative power. KNN exhibited the lowest accuracy (70.31%), performing below all other models.

The model validation parameters indicate that SVM demonstrated the best overall performance (**Table 1**). It achieved the highest values for F1 Score (0.9209), Recall (0.9481), and AUC-ROC (0.9684), suggesting that SVM effectively combines precision and sensitivity, making it the most suitable choice for classifying spectral data from healthy and diseased plants. MLP also performed well, particularly in Recall (0.9111) and ROC-AUC (0.9473), demonstrating strong detection of the positive class.

**Table 1:** Result of the performance evaluation metrics of the models on the test set.

Classification methods	Confusion matrix		Precision	Recall	F1 Score	ROC-AUC
SVM	TP = 128	FN = 7	0.8951	0.9481	0.9209	0.9684
	FP = 15	TN = 106				
MLP	TP = 123	FN = 12	0.8662	0.9111	0.8881	0.9473
	FP = 19	TN = 102				
XGBoost	TP = 109	FN = 26	0.8074	0.8074	0.8074	0.8924
	FP = 26	TN = 95				
RF	TP = 110	FN = 25	0.7692	0.8148	0.7914	0.8493
	FP = 33	TN = 88				
DT	TP = 103	FN = 32	0.7203	0.7630	0.7410	0.7638
	FP = 40	TN = 81				
KNN	TP = 92	FN = 43	0.7360	0.6815	0.7077	0.7607
	FP = 33	TN = 88				

K-Nearest Neighbors (KNN); Decision Tree (DT); Random Forest (RF); Extreme Gradient Boosting (XGBoost); Multi-Layer Perceptron (MLP); Support Vector Machine (SVM); True Positive (TP); False Positive (FP); False Negative (FN); True Negative (TN). Receiver Operating Characteristic - Area Under the Curve (ROC-AUC).

In contrast, XGBoost yielded more consistent results, with a Precision of 0.8074, an F1 Score of 0.8074, and an ROC-AUC of 0.8924, indicating a lower level of discrimination compared to SVM and MLP. RF, while exhibiting a relatively high Recall (0.8148), underperformed relative to the more complex models, with an F1 Score of 0.7914 and an ROC-AUC of 0.8493.

DT displayed limited performance, with lower metrics such as an ROC-AUC of 0.7638. Finally, KNN showed the weakest performance, with a Precision of 0.7360 and Recall of 0.6815, indicating it was the least suitable model for this dataset.

**Figure 4:**
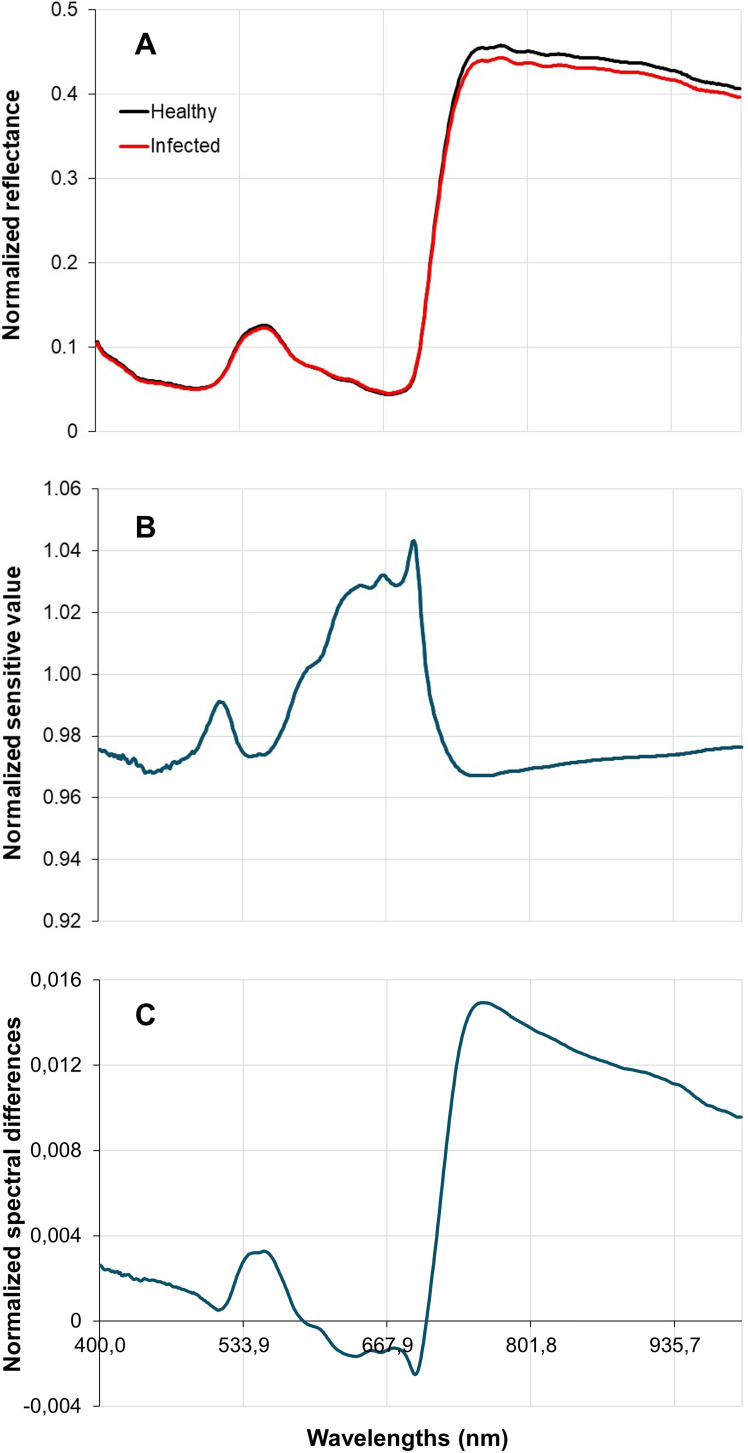
Cassava leaves spectral reflectance characterization: normalized spectral reflectance curves **(A)**; normalized sensitivity value **(B)**; normalized spectral difference **(C)**.

The spectral signature derived from the normalized reflectance of healthy and Xpm-infected leaves is shown in **Figure 4A**. In the near-infrared (NIR) region (700–1000 nm), a difference was observed between healthy and infected leaves. The highest sensitivity was recorded in the 640–700 nm range, which encompasses the red region (640–680 nm), known for chlorophyll absorption (**Figure 4B**). The largest spectral differences between healthy and infected leaves occurred around 760 nm in the NIR range (**Figure 4C**).

## 4 Discussion

The integration of machine learning techniques with hyperspectral data for diagnosing cassava bacterial blight has yielded promising results, particularly with the SVM. This model emerged as the most effective model for distinguishing between healthy leaves and those infected with *Xanthomonas phaseoli* pv. *manihotis*, based on all performance metrics. Following closely was the MLP, which also demonstrated itself as a viable alternative. XGBoost and RF exhibited intermediate performances, while Decision Tree and KNN were the least suitable for this task.

The SVM superiority can be attributed to its capability to manage complex, high-dimensional data, an advantage highlighted in various plant disease classification studies (Nagasubramanian et al., 2018). The use of kernel functions in SVM enables the transformation of data into a higher-dimensional space, which facilitates class separation and mitigates the risk of overfitting (Pathak et al., 2022). The MLP achieved an accuracy that was only 3.52% lower than that of the SVM, showcasing its strong performance in classifying healthy and infected cassava leaves based on hyperspectral data. Its proficiency in processing large volumes of complex hyperspectral data has been corroborated by other studies on plant diseases (Abdulridha et al., 2020; Lee et al., 2022).

XGBoost and RF are ensemble learning models based on decision trees, designed to combine the predictions of multiple decision trees to enhance accuracy, resulting in more robust and reliable outcomes (Breiman, 2001; Chen and Guestrin, 2016; Nti et al., 2023). The adoption of this strategy effectively mitigates overfitting, and despite the distinct characteristics of each model, both approaches yielded satisfactory results. These findings highlights the effectiveness of ensemble techniques in the analysis of spectral data.

The DT and KNN models were considered the least suitable for the classification task, exhibiting low precision and poor generalization. Their limited capacity to process high-dimensional and complex datasets, including hyperspectral data, results in reduced performance (Bramer, 2002; Halder et al., 2024; Zhang et al., 2025). These findings indicate that lower-complexity models like DT and KNN are unable to extract truly meaningful information from such spectral data. Recent research supports this, showing that one-dimensional convolutional neural networks (1D-CNNs) outperform traditional algorithms such as PLS-DA, KNN, and RF in distinguishing rice bacterial blight caused by different pathogens (*Xanthomonas oryzae* pv. *oryzae*, *Pantoea ananatis*, and *Enterobacter asburiae*), due to their more robust architectures that enhance feature extraction and generalization (Zhang et al., 2025).

These differences in algorithm performance indicate that each method has distinct capabilities when processing hyperspectral data for binary classification of healthy and diseased plants. In a previous study, differences in machine learning algorithm performance were observed when comparing hyperspectral data of a fungal disease (*Corynespora cassiicola*) and a bacterial disease (*Xanthomonas euvesicatoria* pv. *perforans*) causing leaf spots in tomato plants, under both benchtop and unmanned aerial vehicle conditions. The multi-layer perceptron (MLP) method achieved higher accuracy values compared to the stepwise discriminant analysis (STDA) method (Abdulridha et al., 2020). Another publication analyzed four fungal diseases in tomatoes (*Botrytis cinerea*, *Fusarium oxysporum*, *Alternaria alternata*, and *Alternaria solani*) using hyperspectral and RGB images with a RF model. Hyperspectral imaging proved more accurate, revealing distinct spectral signatures for effective disease differentiation (Javidan et al., 2024). Therefore, the effectiveness of each technique can be influenced by the nature of the data and the interaction between the plant pathogen and the host.

Spectral data have been widely employed to identify physiological and biochemical changes induced by plant pathogens, providing critical insights into the differences between healthy and diseased plants (Abdulridha et al., 2020; Castro-Valdecantos et al., 2024).

In the present study, higher normalized reflectance values in healthy plants in the near-infrared region (NIR, 700–1000 nm) suggest intact cellular structure, essential for physiological functions (Junges et al., 2020; Mevy et al., 2022). Conversely, the lower normalized reflectance observed in infected leaves indicates structural damage and reduced cellular integrity, typical features of bacterial infections (Zhang et al., 2025). This pattern is reinforced by spectral parameters such as sensitivity and spectral differences, with the most pronounced alterations observed within the near-infrared range.

These findings align with observations from research on the physiological changes induced by *Xanthomonas phaseoli* pv. *manihotis* (Xpm) in cassava leaves. Such investigations revealed a reduction in water potential associated with increased stomatal resistance, along with a rise in proline concentration, indicating the plant’s response to disruptions in cellular homeostasis due to bacterial infection (Rubio et al., 2017).

Similarly, a study on *Xanthomonas citri* subsp. *citri* in Sugar Belle mandarins demonstrated that vegetation indices related to chlorophyll and water content effectively detected early-stage bacterial infections (Abdulridha et al., 2019). In a study utilizing hyperspectral data and vegetation indices to assess healthy and *Xanthomonas euvesicatoria* pv. *perforans*-infected tomato plants, significant physiological differences between the groups were detected as early as two hours post-inoculation. At this stage, spectral bands in the ranges of 740–750 nm and 1404 nm were identified as the most critical for distinguishing between healthy and infected plants (Zhang et al., 2024). Furthermore, the spectral data for the four fungal diseases in tomatoes were most effective in the 500–550 nm and 740–950 nm ranges, which encompass the infrared wavelengths, for early-stage identification and diagnosis (Javidan et al., 2024).

This study advanced the understanding of the hyperspectral behavior of cassava leaves under healthy and infected conditions and demonstrated the potential of integrating this technique with machine learning models for the identification of diseased plants. However, because only one susceptible cultivar was used as the reference for analysis, further studies are needed to assess possible variations in spectral behavior among cultivars.

Furthermore, further research will be necessary to refine this technique for detecting infected propagative material, including cuttings for commercial cultivation and seeds for breeding programs. Additionally, the use of drone-mounted cameras and portable sensors emerges as a potential application of this technique in production fields in the future.

## 5 Conclusions

The integration of machine learning techniques with hyperspectral data has proven effective in detecting physiological alterations caused by cassava bacterial blight in cassava leaves, with SVM achieving the best overall performance. The MLP also exhibited strong performance, while XGBoost and RF produced satisfactory results. The variations in algorithm performance highlight the importance of selecting appropriate methods for the specific pathosystem under evaluation. In addition to that, spectral analysis demonstrated that physiological changes induced by *Xanthomonas phaseoli* pv. *manihotis* can be detected through near-infrared reflectance, reinforcing the significance of spectral techniques in diagnosing plant diseases.

## Data Availability

The raw data supporting the conclusions of this article will be made available by the authors, without undue reservation.
